# Microfluidics-Integrated Spectroscopic Technologies for Food Safety and Quality Assessment: From Complex-Matrix Processing to On-Site Decision-Making

**DOI:** 10.3390/foods15183171

**Published:** 2026-09-08

**Authors:** Jingwen Zhu, Xianjun Sun, Yu Guo, Zhenghao Zhang, Xiaoyan Geng, Hui Jiang

**Affiliations:** School of Electrical and Information Engineering, Jiangsu University, Zhenjiang 212013, China; 2112407124@stmail.ujs.edu.cn (J.Z.);

**Keywords:** microfluidics, spectroscopic analysis, food safety, lab-on-a-chip, optofluidics, point-of-need, machine learning

## Abstract

Food safety and quality analysis is shifting from laboratory-based end-point testing toward faster, lower-volume and matrix-adapted on-site decision-making. Near-infrared (NIR), visible-near-infrared (Vis-NIR), hyperspectral, Raman, surface-enhanced Raman scattering (SERS), fluorescence, colorimetric and terahertz approaches, together with impedance time-series readout, provide complementary information on composition, molecular vibrations, spatial distribution, reaction outputs, or electrical responses. In real foods, however, lipids, proteins, sugars, salts, pigments, particles and native fluorescence can alter spectral baselines, mass transfer and model stability. The value of microfluidics is therefore not limited to miniaturization but lies in organizing filtration, homogenization, splitting, mixing, extraction, enrichment, reaction, and readout positions into a controllable sample-to-signal workflow. This review first distinguishes chemical hazards, biological hazards, authenticity issues, and quality changes according to target and matrix characteristics, and then compares the functional boundaries of continuous-flow, paper-based, droplet, digital-hybrid and enrichment-oriented chips. It further analyses how microfluidics affects detection time, sample and reagent consumption, sensitivity, selectivity, repeatability, portability and cross-matrix applicability through spectral interfaces, signal enhancement, labelled and label-free detection, chemometrics, and machine learning. Representative applications involving pesticides, mycotoxins, pathogens, antibiotics, heavy metals, adulterants, oxidation products, and freshness indicators in real foods are discussed within a unified chain linking chip architecture, spectral signal generation and decision models. Finally, requirements for translation are proposed in terms of standard and real samples, chip-to-chip variation, external model validation, data traceability and scalable manufacturing, providing an operational framework for the joint design of broad-spectrum spectroscopic technologies and microfluidic systems.

## 1. Introduction

The central task of food safety and quality testing has moved beyond the confirmation of single standard solutions in the laboratory. It increasingly requires rapid and reliable judgement of trace hazards, compositional changes, and authenticity differences in real food samples. Pesticide residues, veterinary drugs, mycotoxins, heavy metals and illegal additives usually occur at low concentrations and are unevenly distributed in cereals, fruits and vegetables, dairy products, meat products and edible oils. Freshness, oxidation, maturity and adulteration ratios, by contrast, often change continuously and cannot be reduced to a single spectral peak or a binary threshold. Chromatographic and mass spectrometric methods provide high quantitative accuracy and confirmatory power, but they commonly require complex extraction and clean-up, substantial solvent consumption, centralized instrumentation and long analytical workflows. Sample origin, contamination level, spectral range, preprocessing and independent validation must be reported together; otherwise, the correlation coefficient (R), root mean square error (RMSE) and detection limits are difficult to compare across studies. The practical performance of portable spectroscopy is also affected by sample containers, optical path length, operators and data-transfer procedures.

Spectroscopic technologies offer rapid, minimally destructive and even non-destructive routes for observing foods. Near- and mid-infrared spectroscopy mainly reflect bulk changes in water, protein, lipid, and sugar contents. Raman spectroscopy and surface-enhanced Raman spectroscopy (SERS) provide molecular vibrational fingerprints. Hyperspectral imaging retains the spatial distribution of targets on sample surfaces, whereas fluorescence and colorimetric methods convert specific reactions into imageable or directly readable signals. Nevertheless, a spectral signal is not equivalent to target concentration. Lipids, proteins, pigments, salts, particles and native fluorescence may alter baselines, scattering, absorption, or reaction backgrounds, and SERS is further affected by hotspot heterogeneity, competitive adsorption and fluorescence interference. Chemometric studies of Raman/SERS for food analysis indicate that preprocessing, variable selection, internal standards, and reference methods must be designed together [1]. Studies on nanostructured and flexible substrates also identify substrate uniformity, field sampling and complex backgrounds as major constraints in food applications [2,3]. Thus, the advantages of spectroscopy become comparable analytical performance only when sample states are controlled, readout regions are representative and models are independently validated.

Microfluidics should not be viewed simply as a means of shrinking a detection device. Its role is to organize sample introduction, splitting, filtration, mixing, extraction, enrichment, reaction and optical readout into a reproducible workflow. For liquid foods and extracts, continuous channels can shorten mass-transfer distances and stabilize optical path length in the detection zone. For particulate samples, membrane filtration, sedimentation, or sorting modules can reduce scattering and clogging. For low-abundance pathogens or toxins, immunomagnetic enrichment, aptamer capture or nanomaterial-assisted enrichment can transfer targets into regions more suitable for readout. In paper-based platforms, capillary driving can also reduce dependence on pumps and external connections. Reviews on food Raman/SERS detection have identified pesticides, toxins, antibiotics, and microorganisms as typical application targets [4], while portable SERS and food-safety spectroscopy studies emphasize field sampling, substrate consistency and background control [5,6]. A review of terahertz solution-phase sensing has integrated microfluidic sample handling, metamaterial-enhanced interfaces, and functional materials into a single food-analysis workflow [7]. Non-food microfluidic SERS studies provide transferable experience in magnetic enrichment, dielectrophoretic manipulation, in-channel optical positioning and external calibration [8]. Early applications of portable Raman instruments also show that instrument mobility must be coupled with simple and stable sample contact to support rapid screening [9]. These functions, however, do not automatically lower detection limits, because chips may still suffer from channel clogging, adsorption loss, batch variation and material background.

From the perspective of method selection, food spectroscopy involves at least three coupled layers. The first is whether the sample has been homogenized, filtered, extracted or enriched sufficiently to reduce matrix effects. The second is whether the chip and optical interface can generate stable signals at defined flow rates, reaction times, and detection positions. The third is whether spectral preprocessing and machine-learning models can distinguish target-related differences from variation caused by batch, cultivar, origin, temperature, and instrumentation [10]. For pesticides and mycotoxins, the emphasis is usually on the transfer and selective recognition of low-concentration targets. For authenticity and quality assessment, the emphasis shifts to multi-batch coverage, appropriate reference values and external model stability.

On this basis, this review does not restrict the discussion to SERS. Instead, near-infrared, visible-near-infrared, hyperspectral imaging, Raman/SERS, fluorescence, colorimetric, terahertz, and impedance time-series spectroscopies are compared within a common food analysis framework. The review first discusses analytical requirements determined by food targets and complex matrices, then examines sample introduction, splitting, enrichment, reaction and detection zone design in microfluidic chips. It subsequently compares spectral interfaces and their effects on time, sample consumption, sensitivity, selectivity and repeatability, discusses the role of chemometrics and machine learning in signal interpretation, and uses real-food applications to test the boundaries of method validity. Standardization, external validation, data traceability, scalable manufacturing, and field translation are finally treated as independent issues. The central argument is that the value of microfluidics must be demonstrated as verifiable sample processing and signal control, rather than being summarized only by portability or high sensitivity.

The main aim of this review is to clarify when and how microfluidics improves spectroscopic food analysis in complex matrices. Despite rapid progress in food spectroscopy, SERS substrates, microfluidic assays, and machine-learning-assisted food analysis, several gaps remain. Many spectroscopic studies report analytical performance without clearly specifying how sample state, target transfer and matrix interference are controlled before readout. Conversely, microfluidic devices are often described mainly as miniaturized platforms, although their measurable contributions to filtration, enrichment, reaction control, optical path stabilization, and model reliability are not always separated. Direct validation in real food matrices also remains less abundant than methodological demonstrations in buffers, model systems, or pure spectroscopic datasets. Compared with previous reviews that mainly address food spectroscopy, SERS substrates, Raman/SERS-based food detection, microfluidic assays, or machine-learning-assisted food analysis as separate topics, this review connects these gaps within a single sample-to-signal-to-decision framework. Its contribution is not simply to summarize more spectroscopic routes, but to evaluate how sample introduction, pretreatment, enrichment, detection zone design, spectral modelling, and field judgement interact in real food analysis. Accordingly, Section 2 describes the literature search strategy and evidence-mapping logic; Section 3, Section 4 and Section 5 discuss food targets, microfluidic architectures, and spectroscopic integration; Section 6, Section 7 and Section 8 evaluate performance boundaries, chemometric or machine-learning decisions, and real-food validation; and Section 9 summarizes standardization, traceability and translation requirements.

## 2. Literature Search Strategy

This review was conducted as a selective narrative review with an evidence-mapping purpose. The literature search was performed using Web of Science, Scopus, PubMed, ScienceDirect, Google Scholar and CNKI. The search terms were used alone and in combination, including microfluidics, microfluidic chip, lab-on-a-chip, optofluidics, food safety, food quality, spectroscopy, near-infrared spectroscopy, visible-near-infrared spectroscopy, hyperspectral imaging, Raman spectroscopy, surface-enhanced Raman spectroscopy, SERS, fluorescence, colorimetry, terahertz spectroscopy, impedance spectroscopy, machine learning, chemometrics, mycotoxin, pesticide residue, heavy metal, foodborne pathogen, adulteration, authenticity, and freshness. The main publication period was from 2014 to 2026, while earlier methodological or foundational studies were included when they were necessary for explaining spectroscopic principles, microfluidic architectures or data-analysis concepts. The final search was completed on 28 August 2026.

Studies were included when they met at least one of the following criteria: they reported microfluidic, paper-based microfluidic, optofluidic, or lab-on-a-chip systems coupled with spectroscopic or optical readout for food safety or quality analysis; they provided direct evidence of food or food-related sample analysis using microfluidic–spectroscopic platforms; they described spectroscopic food-analysis strategies needed to explain target categories, matrix interference, calibration, validation, or field translation; or they provided transferable methodological evidence for microfluidic enrichment, optical-interface design, spectral modelling or portable readout. Studies were excluded if they were unrelated to food analysis, lacked spectroscopic or optical readout, provided only general sensor descriptions without analytical validation, or did not contribute to the framework of sample-state control, spectral acquisition, and decision-making. Because this is not a systematic review or meta-analysis, the literature set may still be affected by selection bias. To reduce this risk, studies were selected according to their relevance to food-matrix complexity, microfluidic function, readout mechanism, quantitative performance, validation level and translation potential, rather than according to author group or institution. Some studies from the authors’ group were retained because they provide a continuous research line from conventional spectroscopic acquisition of food information to microfluidic sample-state control before spectroscopic readout, covering near-infrared spectroscopy, Raman/SERS analysis, multimodal spectral modelling and matrix-oriented food detection. Independent studies from other groups were also added or emphasized for SERS, paper-based microfluidics, optofluidics, pathogen detection, food authentication, quality assessment, and field-readable sensing. Non-food methodological studies were retained only when they clarified transferable chip or optical-interface principles, and were not counted as direct food-validation evidence.

## 3. Food Targets, Complex Matrices, and Analytical Performance Requirements

Targets in food safety and quality analysis are hierarchically different. Chemical hazards usually require trace quantification, biological hazards emphasize capture, recognition and viability, or nucleic-acid signals, authenticity problems depend on representative modelling across cultivars and origins, and quality attributes often vary continuously rather than through a single threshold. A single microfluidic structure therefore cannot be assumed to fit all foods. Chip design should first define the initial concentration, physical form, matrix-binding mode and decision basis of the target, and only then determine whether filtration, dissolution, enrichment, reaction, separation or merely optical-path stabilization is required.

### 3.1. Chemical Hazards: Trace Targets and Selective Pretreatment

Pesticides, veterinary drugs, antibiotics, mycotoxins, heavy metals, illegal additives, packaging migrants and processing contaminants do not share the same spectral response. Some pure spectroscopic studies are retained in this section to define matrix-related analytical problems rather than to serve as direct evidence of microfluidic integration. For example, Tian et al. employed visible light near-infrared hyperspectral imaging and the VISSA-SR feature wavelength selection algorithm to conduct non-destructive classification and identification of apples from different origins [11], whereas Arslan et al. developed near-infrared chemometric prediction of antioxidant activity in black goji berries [12]. Together, these studies show that spectroscopy can capture composition- or contamination-related differences, but that signal intensity is not inherently proportional to target concentration. For acetamiprid in green tea, Li et al. used an unmodified gold-nanoparticle SERS/colorimetric strategy [13], whereas Jiang et al. tracked acid value during edible-oil storage using a portable near-infrared system and variable-selection algorithms [14]. In an actual chip, the former must address polyphenol and pigment backgrounds, whereas the latter must handle global spectral changes caused by continuous lipid oxidation. The two tasks therefore require different channel residence times, separation approaches and calibration strategies. The fusion of fluorescence hyperspectral imaging and near-infrared spectroscopy for non-destructive cadmium detection in lettuce further indicates that heavy-metal determination in high-background leaves still requires joint spectral variables and suitable reference labels [15].

In cereals and oils, mycotoxins often occur at low concentrations and with heterogeneous spatial distributions [16]. Liu et al. compared characteristic-wavelength optimization for Fourier transform near-infrared (FT-NIR) determination of aflatoxin B1 in maize [17], Deng et al. further examined how characteristic-wavelength optimization improved aflatoxin B1 prediction in maize [18], and Zhu et al. used wavelength optimization to improve FT-NIR quantification of zearalenone in wheat [19]. These findings show that variable selection can reduce spectral redundancy but cannot replace sample homogenization and target enrichment. For mineral oil contamination and multi-component heavy metals in corn oil, Deng et al. used explainable ensemble learning with FT-NIR spectroscopy [20], and Jiang et al. combined nano-modified colorimetric sensors with near-infrared spectroscopy for heavy-metal detection in corn oil [21]. A review of Raman/SERS models for food hazards also indicates that matching matrix background, feature variables and reference detection is central to quantitative credibility [22].

Pesticide residues are especially challenging because structurally similar compounds may coexist and be heterogeneously distributed on sample surfaces. Yang et al. combined SERS signals with intelligent variable-selection models to detect chlorpyrifos in tea [23], and Zhu et al. also analysed chlorpyrifos residues in tea using SERS and chemometrics [24]. For cabbage and Chinese cabbage, Shi et al. proposed a general model for the in situ detection of pymetrozine residues [25], whereas Zhai et al. used gold nanostars for duplex detection of pesticide residues in grapes [26]. Substrate studies on metal nanowires and nanostars, including chemically processed interfacially assembled nanowires [27] and high-density Ag nanogaps [28], further illustrate the importance of enhancement structures. These studies do not demonstrate that all foods can be measured directly in situ. Rather, they indicate that microfluidic pretreatment should be selected according to target location, using filtration, extraction, magnetic capture, or surface elution as needed, and that substrate enhancement should not be mistaken for solved matrix interference.

Antibiotics, illegal additives, and processing contaminants also involve cross-responses among structurally related compounds. Ahmad et al. systematically discussed sample preparation and signal amplification in antibiotic detection by SERS [29]. Dies et al. detected toxic contaminants in minimally processed foods using dendritic SERS substrates and emphasized sample contact and substrate surface chemistry [30]. Aheto et al. used activated carbon/silver nanoparticle conjugates to capture malathion molecules [31]. For hydrogen peroxide in foods, Li et al. constructed a magnetic nanozyme-based SERS-colorimetric dual-mode assay [32], and Yan et al. analysed nitrofuran residues in honey by SERS [33]. In more field-oriented strategies, Kesama et al. fused NIR and SERS features and reported a pesticide residue quantification model with a prediction R of 0.988 and a residual predictive deviation (RPD) of 8.290 [34]. Nazli Oncer et al. detected pyrimethanil, imidacloprid, and chlormequat chloride in chilli juice [35], showing that mixed-pesticide recognition requires both SERS spectra and unsupervised learning. The supported conclusion is that microfluidic chips should reduce matrix complexity, control mixing ratios and fix sampling volume, rather than being described generically as tools that improve sensitivity.

### 3.2. Biological Hazards: Capture and Judgement at Low Initial Abundance

Foodborne bacteria, viruses, fungi, bacterial toxins, and microbial metabolites share the difficulties of low initial abundance, complex target morphology, and biological variability. Although Tyrophagus putrescentiae and Cheyletus eruditus in flour are not typical microbial pathogens, He et al. used hyperspectral imaging to show that biological foreign matter in particulate foods can generate marked spatial heterogeneity [36]. Conventional multiplex real-time polymerase chain reaction (PCR) can quantify *Escherichia coli* O157:H7 and *Salmonella* spp. in milk [37], while deep-ultraviolet multispectral fluorescence imaging has been used to detect and monitor foodborne pathogens on leafy greens [38]. These findings indicate that detection limits are determined not only by instrumental noise but also by whether targets can be transferred efficiently from food surfaces, suspensions, or extracts to the detection region. A review of Raman spectroscopy-based microfluidic platforms for foodborne pathogens identifies cell capture, labelling reactions, and complex-matrix clean-up as key steps in microfluidic integration [39]. A systematic review on foodborne pathogens and antimicrobial resistance in food microbiomes further supports the view that biological hazard detection is complicated by microbial diversity, matrix-associated variability and the need for reliable target recognition in complex food systems [40].

In pathogen detection, paper-based or elastomeric microfluidic platforms can compress sample allocation, capture, and reaction into small volumes, but the output must still be compared with clear positive/negative criteria or reference methods. Lysozyme on food-contact surfaces can be identified using aptamer-SERS methods [41], and bacterial concentration coupled with SERS readout in drinking water illustrates how channels, filtration, and detection windows can operate together [42]. Point-of-use fluorescence immunoassays further integrate contaminant capture and photoelectric detection on chips [43]. These studies provide engineering guidance for low-abundance targets in foods, but non-food water samples or model proteins should not be treated as direct food validation. In food matrices, proteins, polysaccharides, lipid droplets, and particles can simultaneously affect capture efficiency, washing background, and spectral visibility. Enrichment before readout is therefore often more robust than direct irradiation of untreated samples.

### 3.3. Food Authenticity and Adulteration: Spectral Differences Require Cross-Batch Validation

Authenticity problems include adulteration of edible oils, honey dilution with syrup, water adulteration in dairy products, origin identification of tea and spices, species or geographical-origin discrimination, and formulation authenticity in processed foods. The target is often not a single exogenous molecule, but the composite spectral expression of composition ratios, source differences or processing history. Studies on fumigated and dyed *Lycium barbarum* show that sample origin and processing history can change spectral profiles [44]. Near-infrared spectroscopy combined with chemometrics for goji berries further demonstrates that geographical-origin discrimination and flavonoid-content prediction can be addressed together, but only when origin labels and composition labels are both explicitly defined [45]. Vibrational spectroscopy and chemometrics for dairy adulteration show that changes in protein, water, and non-protein nitrogen may occur together [46]. Models relying on only a few peaks may therefore misclassify natural batch variation as adulteration.

For vegetable oils and beverages, NIR, Raman, Fourier transform infrared spectroscopy (FTIR) and machine learning can support rapid screening, but the role of microfluidics is mainly to make mixing, dilution and splitting reproducible rather than to create new authenticity markers. Wang et al. used NIR spectroscopy and optimized chemometric models for comprehensive quality assessment of Zhenjiang aromatic vinegar [47], and a Vis-NIR transmittance study covering apples from different origins constructed a general model [48]. These studies indicate that authenticity judgement depends less on a single peak than on whether training samples cover variation in origin, variety, formulation, and processing state.

For honey, milk, coffee, tea, hops, and other samples with geographical or formulation characteristics, classification accuracy and interpretability must be considered together. Poursorkh et al. detected non-protein nitrogen adulterants in milk using Raman spectroscopy and machine learning [49]. Fanning et al. used near-infrared multivariate analysis to trace the geographical origin of New Zealand hops [50]. El Orche et al. compared vibrational spectroscopy machine-learning classification methods for geographical-origin determination of raw milk [51]. Diffuse reflectance infrared Fourier transform spectroscopy (DRIFTS) detection of multiple adulterants in roasted coffee [52], FTIR pattern-recognition detection of coffee adulteration [53] and visible-near-infrared (Vis-NIR) prediction of bioactive compounds in edible flowers [54] further show that quantitative adulteration, composition prediction and origin classification have different data structures. If future chips are to serve authenticity assessment, they should prioritize representative sampling, homogeneous mixing, and cross-batch calibration rather than single-test classification accuracy. Ehsani et al. identified water adulteration in milk using portable NIR spectroscopy with ensemble classification and regression [55], and Pan et al. used NIR spectroscopy to determine the geographical origin and storage age of tangerine peel [56]. Such studies indicate that adulteration, origin and storage state require separately defined labels and cross-batch validation.

### 3.4. Food Quality and Freshness: From Static Indicators to Dynamic Changes

Food quality and freshness usually change continuously through lipid oxidation, protein oxidation, spoilage, maturity, processing variation, and freeze–thaw damage. Long-term NIR evaluation of soluble solids in apples [57], polarized spectra and hyperspectral fusion for soluble sugar and total nitrogen in greenhouse tomato leaves [58], and Raman quantification of edible-oil acid value show that quality indicators are affected by sample composition, measurement time and instrument status. Simultaneous quantification of chemical constituents in matcha [59], fluorescence hyperspectral visualization of protein oxidation in freeze–thawed pork [60], and Raman-deep-learning analysis of heat-induced pork batter quality [61] further indicate that spectra can capture global changes caused by processing and storage. To interpret such changes as specific quality attributes, however, independent physicochemical or sensory reference values are required. Huang et al. used a colorimetric sensor based on porphyrin and pH-indicator ligands to monitor pork freshness [62], whereas simultaneous NIR hyperspectral determination of protein, starch, and moisture in wheat flour [63] illustrates that no single chip or spectral window can cover all quality variables. A review of Vis-NIR spectroscopy for characteristic fruit quality detection also emphasizes that portable, online, and vehicle-mounted systems must be calibrated to fruit structure and multi-attribute quality variation [64]. NIR-based chemometric modelling of sauce-flavour Baijiu stacking fermentation further illustrates that process-round information and physicochemical indicators can be integrated for the monitoring of complex fermented foods [65].

Maturity, freshness and storage damage have clear spatiotemporal features. Visible-near-infrared transmittance spectroscopy coupled with machine learning has been used for pineapple maturity classification [66]. Diffuse-reflectance and fluorescence hyperspectral imaging during cold storage of fresh-cut pineapple simultaneously predicted L*a*b*, pH and soluble solids [67]. Multi-indicator hyperspectral imaging and attention mechanisms for freeze–thawed chicken breast placed several quality indicators into a single prediction framework [68]. NIR hyperspectral detection of protein, starch and moisture in flour [63], modelling of tea withering [69] and vision-spectral modelling during drying [70] show that process monitoring requires stable sampling positions and time synchronization more than end-point judgement does. NIR measurement of mass flow rate in a grain vibration-feeding system also indicates that fluid transport, particle movement and spectral acquisition are coupled [71]. In microfluidic systems, this coupling appears as joint variation in flow rate, residence time, and detection-window geometry.

Quality assessment should not be judged only by model. Spatial variation in fresh-cut fruits and vegetables, fat and moisture in meat, pigments in tea, and viscosity in oils can all cause model failure in new batches. A review of edible-oil evaluation notes that NIR, mid-infrared (MIR) and Raman have distinct advantages in composition, oxidation, and adulteration assessment [72]. A review of fish quality and safety analysis also emphasizes that spectral models should correspond to reference indicators such as microbial load, total volatile basic nitrogen, and lipid oxidation [73]. The appropriate role of microfluidics in quality analysis is therefore to stabilize sample volume and optical path, reduce particle sedimentation or oil–water separation, and, when necessary, perform dilution, mixing and time control on chip. Dynamic quality changes should not be simplified into a one-off pass/fail decision.

### 3.5. Interference Sources in Complex Food Matrices

High fat, high protein, high sugar, high particle loading, high viscosity, high salt, strong pigments, native fluorescence, and multi-component coexistence repeatedly interfere with food spectroscopy. Proteins, lipids, and water in dairy products and milk powder jointly alter near-infrared absorption. Particle size and moisture in grains and flours affect scattering. Pigments and polyphenols in tea, fruits, vegetables and spices increase fluorescence backgrounds. Oils introduce high viscosity, phase separation and unstable optical paths. Reviews on the near-infrared detection of hazards in milk and dairy products [74], mid-infrared spectroscopy combined with chemometrics for agri-food quality evaluation [75], and deep-learning regression in near-infrared spectroscopy [76] all indicate that preprocessing, variable selection, and external validation must be discussed together with matrix effects.

SERS is not inherently immune to matrix interference. A review of flexible SERS substrates emphasizes that sample spreading, substrate contact, and surface contamination affect hotspot utilization [77]. SERS detection of fructose and pectin in fruit foods indicates that natural macromolecules may compete for adsorption on metal surfaces or alter the local environment [78]. Microfluidics can select modules according to matrix characteristics. Membrane filtration is suitable for particle removal, liquid–liquid or solid-phase extraction can reduce lipids and pigments, immunomagnetic beads can transfer low-concentration proteins or bacteria out of the background, and paper-based capillary channels are suitable for small-volume rapid reactions. Each module should have measurable quality indicators, such as filtration loss, enrichment factor, recovery, elution volume, and batch variation. Final spectral peak intensity alone is insufficient to judge whether a chip is effective.

### 3.6. Analytical Performance Metrics

In the following discussion, microfluidic spectroscopic platforms are evaluated using detection time, sample and reagent consumption, limits of detection and quantification (LOD/LOQ), recovery, selectivity, quantitative accuracy, precision, chip repeatability, throughput, portability, automation, and cross-food-matrix generalizability. For contaminants, LOD/LOQ must specify the calibration matrix, blank, replicate measurement, and recovery in real samples. For authenticity and quality tasks, classification accuracy, RMSE, external validation, and the origin of misclassified samples should be reported. SERS studies on food safety in the Oltrepo Pavese area considered regional samples, contamination risk, and sensing methods within the same scenario [79], reminding us that high sensitivity is not equivalent to field usability. These metrics are mapped below to chip architecture, spectral interfaces and model decisions rather than directly comparing performance values across unlike tasks. Table 1 summarizes the food targets, representative matrices, major analytical bottlenecks, and microfluidic intervention points discussed in this section, covering chemical hazards, biological hazards, authenticity/adulteration issues, and quality/freshness changes. It emphasizes that different tasks require different pretreatment and readout strategies.

## 4. Microfluidic Architectures: From Sample Entry to Readable Signals

**Figure 1:**
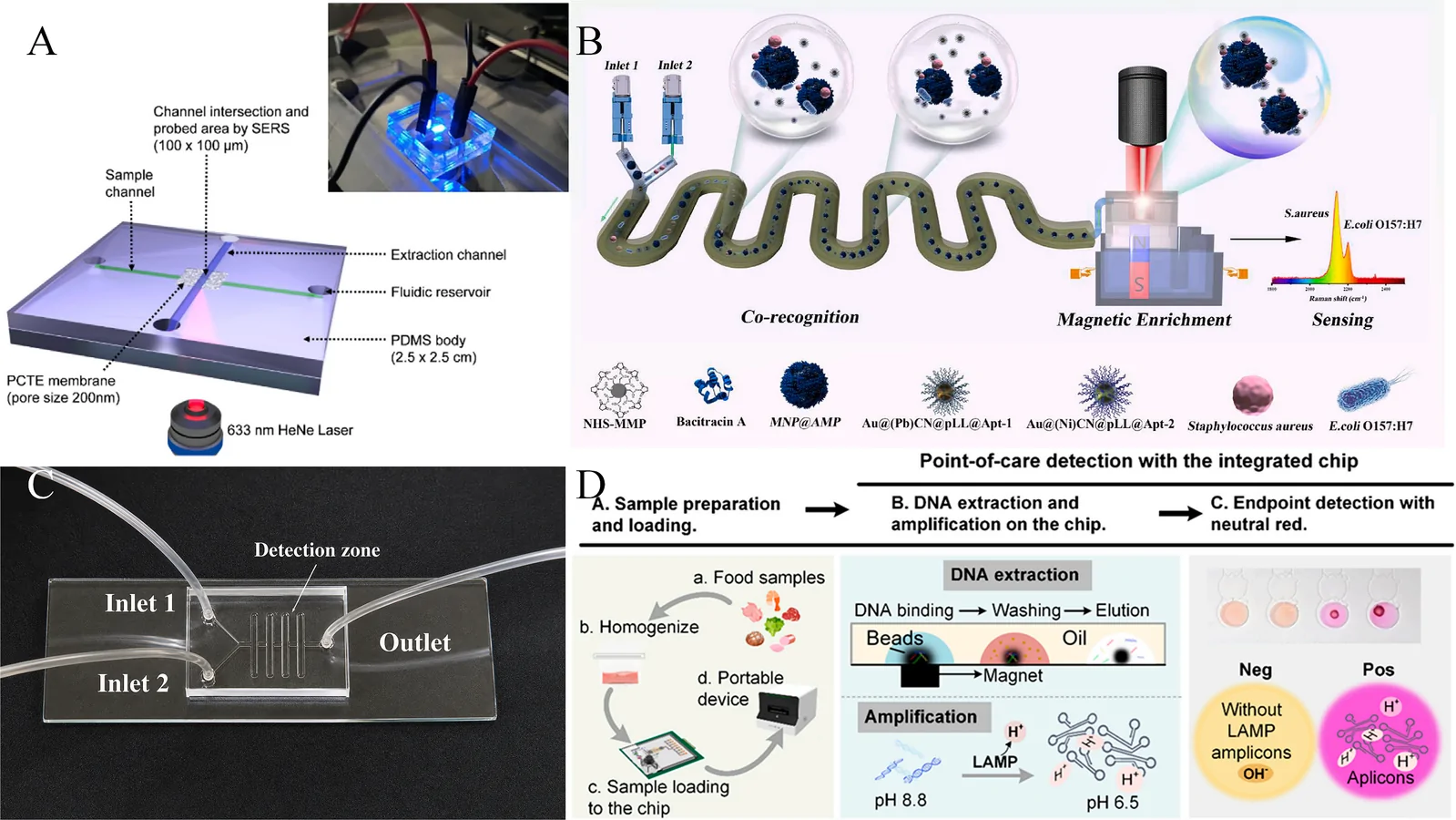
Microfluidic functional modules and the sample-to-signal workflow. (**A**) Microfluidic SERS chip design for sample delivery, membrane-assisted concentration and localized optical interrogation. (**B**) Point-of-care workflow integrating co-recognition, magnetic enrichment, and SERS sensing for bacterial detection. (**C**) Dual-inlet microfluidic channel with a defined detection zone for controlled sample introduction and spectral readout. (**D**) Portable chip-based workflow coupling sample loading, nucleic acid extraction/amplification, and colorimetric endpoint judgement.

The core of a microfluidic architecture is not making channels as small as possible, but delivering samples to the spectral detection zone in a reproducible state. For food samples, chips often need to place several functions between sample inlet and optical readout, including homogenization, filtration, splitting, mixing, enrichment, washing, reaction, drying or quantitative transfer. Particles, bubbles, viscosity changes, and non-uniform sedimentation cause flow fluctuations and can turn spectral measurement from a chemical problem into a geometrical one. A near-infrared study of mass flow in a particulate feeding system demonstrates that transport state itself affects signals [71]. Microfluidic design should therefore optimize flow rate, channel volume, residence time, detection window, and beam size as a single system. Figure 1 outlines the most common functional modules in the microfluidic sample-to-signal workflow, including sample splitting, enrichment/capture, on-chip reaction or SERS readout, nucleic acid extraction, and portable end-point judgement. It provides an overall framework for understanding the subsequent discussion of chip architectures.

### 4.1. Sample Introduction, Splitting and Pretreatment

Sample introduction determines whether subsequent results are representative. Continuous-flow chips are suitable for liquid extracts and cell suspensions. Paper-based chips reduce the need for external pumps through capillary action. Digital or channel-hybrid chips are suitable for dividing one sample into multiple parallel reaction units. For SERS systems that require direct generation of nanomaterials inside chips, 3D-printed microfluidic devices can provide both structural shaping and liquid transport, although surface roughness, residues and dimensional accuracy require separate validation [87]. Spatially offset Raman measurements in 16-micrometre-deep microchannels separately collected signals from liquid and polydimethylsiloxane (PDMS) layers [88], showing that channel material and optical focus must be considered together. Food chips should therefore first ensure that inlets do not clog, fluids do not bypass the intended path, detection-zone thickness remains stable, and each module has an explicitly defined function in the design drawing.

For wheat, maize, dairy products, and edible oils, conventional pretreatment generally includes grinding or homogenization, weighing, solvent extraction, centrifugation or filtration, concentration and further purification. Quick, easy, cheap, effective, rugged, and safe (QuEChERS) extraction, liquid–liquid extraction, and immunoaffinity columns are suited to different pesticides, mycotoxins, and protein targets, but repeated transfers increase solvent consumption, target loss, and batch variation. Microfluidics can compress mixing, splitting, membrane filtration, magnetic capture, and elution into microlitre volumes, while fixed channel volumes control residence time. Rapid on-chip processing, however, cannot replace chromatographic or mass-spectrometric methods required for regulatory confirmation. A more appropriate role is to reduce pretreatment burden, improve the consistency of sample transfer, and lower matrix background before spectral readout.

### 4.2. Enrichment, Separation, and Target Transfer

Low-concentration targets usually require local enrichment before spectral acquisition. Microfluidics can alter the spatial distribution of targets through membrane filtration, dielectrophoresis, magnetic particles, immunocapture, or surface adsorption. Studies on the online preparation of traditional SERS substrates show that laser-induced nanoparticle deposition can form measurable active regions inside channels [89]. Tunable freeform reflectors link reflection geometry with channel position, allowing optical paths to adapt to chip structures [90]. Microsphere-lens arrays embedded in microfluidic chips emphasize that sensitivity and stability require simultaneous control of particle position and optical focusing [91]. Integrated microfluidic-SERS analysis of serum extracellular vesicles [92], mixing-channel design for fluorescence detection of monoclonal antibody (mAb) aggregates [93], and dielectrophoresis-SERS sensing of cancer cells [94] all indicate that capture followed by positioning is a key part of the microfluidic–spectral interface, not a negligible preliminary step.

Cross-disciplinary examples such as the SERS detection of explosives and drop-coating deposition Raman spectroscopy of liposomes [95] show that surface enrichment and sample spreading can change signal visibility. These are not food validations, but they help explain why the same substrate can behave differently in different food extracts. Enrichment modules in food microfluidics must therefore report target loss, nonspecific adsorption, and elution efficiency, and connect these data with recovery in real samples.

### 4.3. Mixing, Reaction and Time Control

Microscale flow shortens diffusion distance, but it does not make all liquids mix rapidly. Serpentine channels, split-and-recombine structures, droplets, pneumatic valves and electrowetting structures suit different viscosities, reaction times and throughput requirements. If clustered regularly interspaced short palindromic repeats (CRISPR), loop-mediated isothermal amplification (LAMP), immunoreactions, or in situ nanoparticle generation are required inside a chip, insufficient mixing can cause false negatives, whereas excessive residence time can increase background. Microfluidic paper-based CRISPR/Cas-SERS studies place pathogen recognition and signal conversion within a single workflow. Channel–digital hybrid platforms for foodborne pathogens integrate sample splitting, parallel reactions, and on-site judgement. These examples show that channel structure evaluation should extend from whether flow occurs to mixing efficiency, reaction uniformity, valve reliability, and end-point time.

### 4.4. Spectral Detection Zones and Chip Materials

A spectral detection zone must balance fluid sealing, material background and optical accessibility. PDMS is easy to process, transparent and suitable for rapid prototyping, but it can adsorb hydrophobic molecules and generate its own Raman background. Glass and quartz have lower optical backgrounds, but increase fabrication and bonding costs. Paper-based materials are inexpensive and disposable, but fibre structure and wetting state can change scattering. The microfluidic spatially offset Raman spectroscopy (SORS) study by Matthiae et al. showed that focus position and spatial offset can distinguish channel liquid from the PDMS substrate [88]. Therefore, the spectral window cannot be determined only by microscope working distance. For portable readout, reflectors, lens arrays, fixed beam spots and chip holders should be considered during the design stage.

### 4.5. Chip Formats and Application Boundaries

Continuous-flow formats are suited to online monitoring and stable sample flow. Paper-based formats suit low-cost, low-reagent field screening. Droplet and digital–hybrid formats facilitate multi-condition reactions and parallel analysis. Dielectrophoretic or magnetic-enrichment formats are more suitable for cells, particles, and low-abundance targets. Microarray formats can increase throughput but require stricter positioning and batch consistency. Studies of microfluidic fluorescence sensing, microfluidic SERS, fluorescence dye mixing and other methods show that format selection must follow target morphology and readout requirements rather than chip appearance. These dimensions together form the framework for choosing chip formats. SERS-enabled lateral-flow assays for food contaminants provide a closely related format example, in which capillary transport, Raman nanotags, and portable readout are integrated, but nanotag morphology and multiplex calibration remain important sources of variability [96].

### 4.6. Principles for Selecting Microfluidic Architectures

From an engineering perspective, architecture selection should be driven by four questions: whether the sample requires particle or lipid removal, whether the target requires active capture, whether the reaction requires precise timing, and whether the spectrum requires a fixed interface and optical path. For liquid foods, continuous flow combined with membrane or magnetic enrichment usually supports more stable transfer from sample to detection zone. For particulate foods, homogenization and extraction volume should be treated as upstream metrics. For pathogens and nucleic acids, capture, lysis, washing, and amplification should be prioritized in sequence. For SERS, the positions of nanomaterials, target molecules and laser focus must be controlled simultaneously. True chip performance is determined by the multiplied losses and fluctuations of each module, not by a single optimal point. Table 2 further compares continuous-flow, paper-based, droplet/digital, dielectrophoretic/magnetic-enrichment, and microarray formats in terms of throughput, sample consumption, enrichment capability, equipment dependence, and compatibility with real foods, avoiding classification based only on chip appearance.

**Table 2:** Comparison of microfluidic formats for spectroscopic food analysis.

Microfluidic Format	Core Function	Main Performance Gain	Main Limitation	Compatible Spectroscopy	Evidence Type and Representative Sources
Continuous-flow/flow-through	Transport, mixing, dilution, online reaction, and readout	Shorter contact/reaction time; supports online monitoring and throughput	Requires pumping or flow control; clogging, and bubbles affect stability	Raman/SERS, NIR, fluorescence	[8,85,89,97]; Figure 2A
Capillary-driven/lateral flow	Capillary-powered loading, transport, and reaction	Pump-free, low cost, low power, field friendly	Flow rate depends on material, viscosity, humidity, and temperature	SERS, fluorescence, colorimetry	[98]
Paper microfluidics	Wetting, filtration, reaction, colour development, and imaging	Disposable, low sample volume, easy storage, phone/handheld compatible	Evaporation, batch variation, and matrix tolerance require control	SERS, fluorescence, colorimetry	[82,83,84,99]
Digital/hybrid microfluidics	Droplet partitioning, programmed operations, and multistep integration	Automation, low cross-contamination, and sample-to-answer workflows	More complex actuation and packaging; higher cost	Fluorescence, colorimetry, SERS	[100]; Figure 1D
Membrane separation/electrokinetic enrichment	Size sieving, electrokinetic migration, and concentration	Raises local target concentration and improves low-abundance sensitivity	Membrane fouling, voltage conditions, and matrix compatibility	SERS, fluorescence	Direct microfluidic SERS methodological evidence: [101]; non-food methodological references retained only for transferable enrichment principles: [42,94]; Figure 1A and Figure 2C
Multichannel/microarray	Parallel reactions and multiple detection zones	Higher multiplexing, throughput, and cross-target comparability	Channel crosstalk, calibration, and readout consistency	Fluorescence, colorimetry, SERS	[82,100]; Figure 1C,D
3D-printed/structured chips	Rapid construction of complex channels and optical windows	Shorter prototyping cycle and customizable channels	Surface roughness, material compatibility, and scale-up remain open	SERS, colorimetry	[87]; Figure 2B

**Figure 2:**
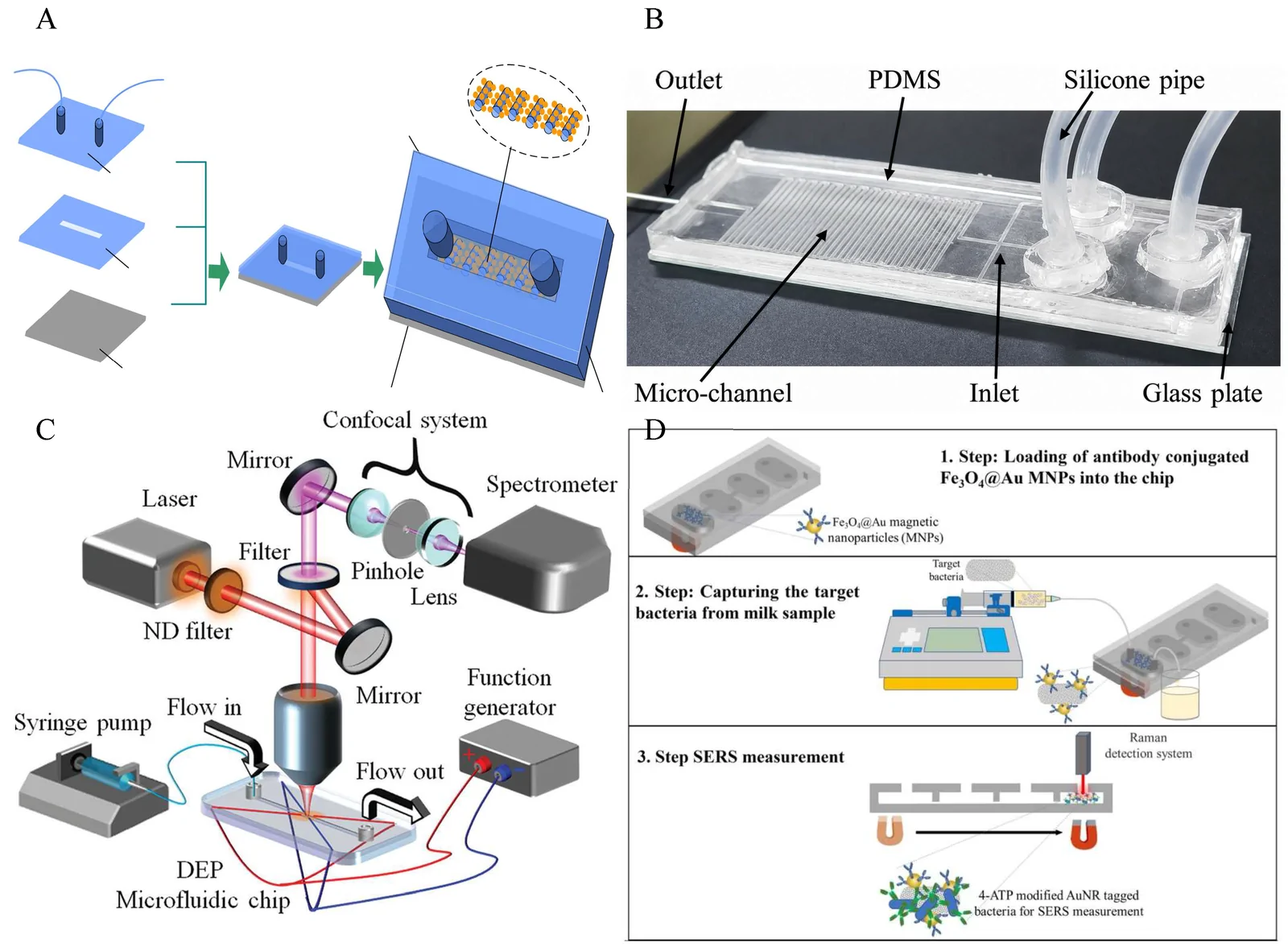
Integration strategies for microfluidic SERS platforms. (**A**) Chip assembly and confined formation of SERS-active nanostructures within the detection region. (**B**) PDMS-based microchannel device configured with inlet, outlet, and optical detection paths. (**C**) Dielectrophoresis-assisted microfluidic SERS setup combining fluidic transport, electric-field enrichment, and Raman acquisition. (**D**) Immunomagnetic capture and on-chip SERS measurement workflow for target-bacteria detection.

## 5. Integration Strategies for Spectroscopy and Microfluidics

The integration of microfluidics and spectroscopy can be grouped into four modes: direct readout inside channels, in situ generation of enhanced substrates on chip surfaces or in detection zones, enrichment inside the chip followed by transfer to an external substrate, and multispectral or multimodal readout. Different spectroscopic routes impose different requirements on channel materials, optical path length, flow rate, and detection-zone design. NIR and hyperspectral approaches rely more on stable geometry and scattering conditions. Raman/SERS requires contact between targets and surfaces or enhanced regions. Fluorescence and colorimetry depend on reaction time and background control. Terahertz sensing must account for water absorption and the dielectric response of functional materials. These differences define the basis for comparing spectroscopic routes and microfluidic interfaces.

### 5.1. Near-Infrared, Hyperspectral and Spatially Resolved Readout

Near-infrared and hyperspectral technologies are well suited for rapid assessment of bulk food composition, spatial distribution, and state changes, but their direct sensitivity to trace targets is often limited. In a wheat mycotoxin study, Zhu et al. coupled solubility-difference-driven microfluidic sorting with near-infrared detection, with the central aim of changing the spatial relationship between targets and matrix before spectral quantification [80]. The prediction-set R values for four mycotoxins exceeded 0.90, and the cost per chip was below 1.25 US dollars (USD). Matthiae et al. used hyperspectral spatially offset Raman spectroscopy in a microfluidic channel and exploited spatial offset and focus adjustment to distinguish superficial liquid from the PDMS layer, while discussing concentration-measurement consistency in small-volume haemoglobin solutions [88]. Non-chip studies, such as those of the near-infrared prediction of antioxidant activity in black goji berries [12] and Raman quantification of edible-oil acid value [102], also demonstrate how spectra respond to global quality changes. However, such results cannot replace microfluidic control of sample state and optical path. Microfluidics can therefore process samples and also act as part of the optical layer structure.

### 5.2. Raman/SERS: From Flow Contact to Enhanced Hotspots

SERS integration usually follows three routes: preloading substrates inside channels so that samples pass through hotspots, generating nanoparticles in situ at channel intersections or detection zones, or enriching targets first with magnetic beads, aptamers, or immunoreactions and then collecting Raman signals in fixed regions. SERS detection of thiabendazole residues on fruit and vegetable surfaces suggests that surface contact can shorten pretreatment, but its transferability depends on whether the target is located on an accessible surface [103]. Portable Raman detection of high-priority drugs illustrates the value of instrument mobility while reminding us that non-food spectral backgrounds cannot be directly extrapolated to foods [104]. Bowtie metal–insulator–metal (MIM) substrates combined with machine learning for multiple pesticide recognition in chilli juice show that enhancement structures, target–metal interactions and model classification must be evaluated together [35]. Reviews of SERS substrates and food Raman applications further indicate that detection-zone area, hotspot uniformity, and internal-standard design directly determine quantitative repeatability [105]. Figure 2 highlights the major integration strategies for microfluidic SERS, including in-channel nanoparticle generation, flow-channel detection zones, dielectrophoretic or magnetic enrichment, and optical focusing/enhancement. It shows that SERS performance is jointly controlled by materials, transport and optical configuration.

### 5.3. Fluorescence, Colorimetric and Impedance Time-Series Readout

Fluorescence and colorimetric readouts require relatively simple instrumentation and are compatible with paper-based chips and portable imaging, but they are susceptible to food colour, native fluorescence, pH, and reagent-batch variation. Although the Vis-NIR prediction of bioactive compounds in edible flowers is not a microfluidic study, it shows that natural pigments and compositional changes can alter multiple spectral variables at the same time. Fluorescence chips therefore need blanks, internal standards, or ratiometric channels. Studies on microfluidic fluorescence immunoassays, paper-based molecular imprinting and cell paper-based chips indicate that detection-zone geometry and exposure conditions of smartphones or portable readers are also important. Deep-learning-enhanced paper-based fluorescence sensing for pesticide residues, together with ratiometric fluorescence paper-based chips for nickel ions and ethylenediaminetetraacetic acid (EDTA) in foods, illustrates feasible paths for multi-target reactions and intelligent handheld interpretation. Because impedance time-series readout does not provide molecular vibrational spectra, it is discussed here as an electrochemical/electrical readout route that can be integrated with microfluidic sample handling, rather than as an optical spectroscopic modality.

### 5.4. Optical, Chemical, and Multimodal Interfaces

Microfluidic platforms can place different signal routes in different regions of the same chip or perform sequential readouts on one sample. Feature-level fusion of NIR and SERS has been used for the quantitative detection of pesticide residues in foods [34], and SERS-colorimetric dual-mode nanozymes have been used for hydrogen peroxide detection [32]. In broader spectroscopic integration, a chip may first use absorption or fluorescence for rapid screening and then use Raman/SERS for structural confirmation. Terahertz functional materials may also enhance liquid-phase samples, but water absorption, temperature, and material stability must be addressed. Chen et al. combined colorimetric, fluorescence, and SERS signals in a trimodal sensor for mercury ions in foods [106], showing that multimodal interfaces can cross-constrain matrix interference. The advantage of multimodality is not simple signal addition. It lies in using the distinct sensitivities of different technologies to matrix and target properties, thereby reducing blind spots of a single spectral route. Terahertz sensing is retained within the scope because recent food-analysis work links solution-phase sensing, microfluidic confinement and metamaterial-enhanced interfaces.

### 5.5. Labelled and Label-Free Detection

Labelled detection uses antibodies, aptamers, enzymes, or molecularly imprinted materials to convert targets into measurable signals. It usually provides stronger selectivity but requires reagent loading, reaction and washing. Label-free SERS, Raman or NIR directly uses the spectrum of the target or matrix change, reducing steps but increasing vulnerability to background and structurally similar interferents. Flow-through aptamer-SERS detection of aflatoxin B1 in food crops represents the specific-capture plus enhanced-readout route. In situ nanoparticle generation inside microfluidic chips for *E. coli* detection in foods represents a shortened operational chain that maintains signal enhancement. For pathogens, combining CRISPR/Cas-SERS with paper-based chips links nucleic acid specificity with spectral fingerprints. Whether a labelled strategy should be used depends jointly on target concentration, matrix complexity, allowable analysis time and field reagent conditions.

### 5.6. Engineering Requirements for Spectral Interfaces

Engineering a spectral interface includes optical-window materials, detection-zone positioning, laser focus, scattering background, detector working distance, and data-acquisition protocol. In SERS, nanoparticles or substrates must coincide with both the focus and the fluid interface. In NIR and hyperspectral imaging, sample thickness, scattering path, and field of view should remain stable. In fluorescence and colorimetry, exposure, white balance, and colour correction affect the result. Chip materials, tubing and sealants used in absorption and Raman routes may also contribute background peaks. Solutions based on quartz, PDMS, paper-based materials and microlenses indicate that optical interfaces should be designed together with chip structures rather than added after a chip has been fabricated. Table 3 compares different spectroscopic routes in terms of signal origin, microfluidic coordination, data interpretation, and applicable food-analysis tasks, emphasizing that spectral interfaces should be designed together with sample state, detection-zone materials and model validation.

**Table 3:** Spectroscopic routes, microfluidic coordination and data-interpretation requirements.

Spectroscopic Route	Primary Information	Microfluidic Synergy	Interpretation/Model	Strengths and Boundaries	Food Tasks and Sources
NIR	Bulk composition, characteristic bands, rapid quantification	Sorting and homogenization; lower particle/matrix heterogeneity	PLSR, wavelength selection, outlier detection	Fast batch screening; limited trace sensitivity and overlapping bands	Wheat mycotoxins; [80], Figure 3A
Raman/SERS	Molecular fingerprints and enhanced trace signals	Hotspot construction/in situ synthesis; magnetic or DEP enrichment; online acquisition	Peak intensity, internal standard, PCA/PLS, classification	High sensitivity and specificity; reproducibility and fluorescence need control	Bacteria, AFB1, contaminants, MDA; [85,89,97,98,101,107,108]
Fluorescence/chemiluminescence	Intensity, ratios, multiplex zones, reaction kinetics	Paper reaction zones, capillary transport, parallel zones	Ratiometric correction, AI imaging, classification/regression	Low cost and portable; probe stability, bleaching and background need control	Veterinary drugs, metals, EDTA, pesticides; [43,82,84,106,109]
UV-vis/colorimetry/SPR	Absorbance, colour, refractive index, endpoint signal	On-chip amplification, reaction control, fixed optical path, phone readout	Thresholding, colour-space analysis, simple classification	Best for field screening; weaker quantitative and multiplex resolution	Pathogens and chemical contaminants; [32,43,100,106]
Multispectral diffraction/imaging	Diffraction fingerprints, morphology, spatial distribution	Target fixation, optical control, channel positioning, image acquisition	Wavelength selection, clustering, classification, deep learning	Label-free potential; transfer, alignment, and real-food validation need work	Pathogenic fungi; [110], Figure 3B
FTIR/infrared/hyperspectral	Functional groups, composition change, defects, spatial heterogeneity	Compartmental sampling, thin-layer control, stable optical path, spatial positioning	PCA, PLS, SVM, CNN, joint spatial-spectral analysis	Rich information; water, background, and instrument size limit deployment	Food composition, quality and authenticity; [54,63,67,68,69,76,81]
Terahertz spectroscopy	Low-frequency molecular vibration, dielectric response and water-sensitive absorption	Microfluidic confinement, thin-layer control, metamaterial-enhanced sensing interfaces, and reduced sample volume	Multivariate calibration, resonance-shift analysis, and background correction	Useful for liquid-phase or high-water food analysis, but water absorption, temperature sensitivity and device complexity limit broad deployment	Food-analysis and microfluidic terahertz sensing; [7]
Impedance time-series spectroscopy	Electrical response related to enzymatic inhibition, ionic transport or interfacial changes	Multilayer paper channels, controlled reagent contact, and fixed reaction volume	Time-series features, threshold judgement and classification/regression models	Suitable for low-cost field screening and pesticide-residue identification, but it is not a vibrational spectroscopic method and requires careful separation from optical spectroscopy	Paper-based microfluidic pesticide-residue identification; [83]

**Figure 3:**
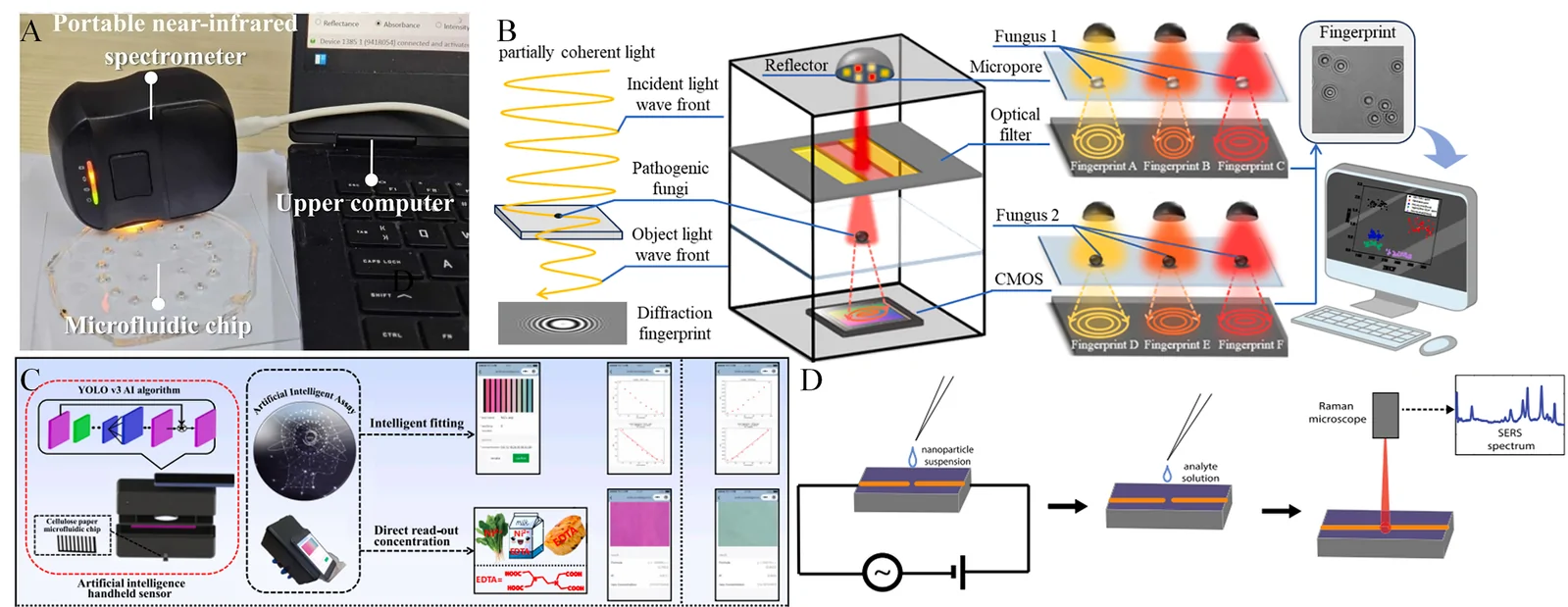
Optical readout and intelligent interpretation routes in microfluidic food analysis. (**A**) Portable near-infrared spectroscopy coupled with a microfluidic chip for field-oriented measurement. (**B**) Lensless or spatial optical fingerprinting strategy for identifying pathogenic fungi through diffraction or image-derived features. (**C**) Fluorescence-based artificial-intelligence readout using image recognition and handheld concentration output. (**D**) Direct SERS readout route based on controlled analyte delivery and localized Raman spectral acquisition.

## 6. Performance Improvements and Boundaries of Microfluidics

The effect of microfluidics on performance should be separated into measurable components: shortening sample-processing time, reducing sample and reagent consumption, increasing the local concentration of targets at the detection zone, lowering background, stabilizing optical path length, improving parallel throughput, or making instruments and workflows more suitable for field use. Microfluidics does not automatically improve every metric. Channel clogging, surface adsorption, chip-batch variation, nanomaterial heterogeneity, and pump fluctuations may offset theoretical sensitivity gains. This section therefore focuses on improvement mechanisms and boundaries and explicitly treats non-food microfluidic or spectroscopic studies as methodological references rather than evidence of food application.

### 6.1. Time, Sample Consumption, and Throughput

The volume scale of microfluidics can reduce reagent and sample consumption and arrange multiple steps on one chip, but total analysis time also includes homogenization, enrichment, washing, reaction, and readout. Deep-learning modelling of tea drying [70] indicates that time-series data are valuable only when sampling rhythm and state annotation are controlled. Fluorescence hyperspectral and NIR fusion for cadmium in tomato leaves [111] shows that adding information under complex backgrounds does not necessarily replace effective sample selection. For microfluidic platforms, reports should include sample-to-answer time, actual sample volume per measurement, waste volume, and number of parallel channels, rather than only single-spectrum acquisition time.

### 6.2. Sensitivity, Selectivity, and Substrate Stability

SERS substrates and enrichment materials can reduce apparent detection limits, but recovery and false positives in real foods better reflect method value. FTIR pattern-recognition detection of coffee adulteration [53] and spectroscopic adulteration recognition in turmeric powder [112] show that selectivity arises from the combined design of signal, variables and samples. A review of hydrogel SERS substrates links material pore structure, target diffusion and anti-matrix capability [113]. Chips should therefore compare blanks, spiked samples, negative matrices, structurally similar interferents, and multiple substrate batches. High signals obtained only in buffer should not be used to claim direct food applicability.

### 6.3. Repeatability, Cost, and Cross-Disciplinary Borrowing

Batch-to-batch repeatability is critical for practical microfluidic spectroscopic systems. Automated infrared/Raman sorting of waste textiles [114], Raman spectroscopy of handmade-paper analysis [115], deep-ultraviolet Raman identification of black plastics [116], and portable SERS detection of high-priority drugs are not food studies, but they all show that material colour, surface state, and complex backgrounds can change model boundaries. SERS studies of explosives [117] provide spectroscopic methodology for trace-target recognition, but should not be written as food validation. For food chips, cost assessment should include moulds, membranes, tubing, nanomaterials, pumps, light sources, and disposable consumables. Portability should not be judged only by instrument weight, but also by calibration, dark-current correction, temperature control, and the need for trained operators.

## 7. Spectral Preprocessing, Chemometrics, and Machine-Learning Decisions

The path from spectra to food decisions usually includes denoising, baseline correction, scatter correction, normalization, feature selection, dimensionality reduction, classification or regression, and external validation. Machine learning can handle complex variables and nonlinear relationships, but it cannot compensate for incorrect sample labels, changing chip states, or unrepresentative training sets. In microfluidic systems, chip-batch variation, flow rate, reaction time, and detection-zone position may become hidden variables recognized by the model. If the training set covers only one chip or one food batch, the model may easily learn device differences as if they were food differences.

### 7.1. From Variable Selection to Model Interpretation

Comprehensive learning particle swarm optimization-support vector machine (CLPSO-SVM) classification of tea quality [118], classification of *Lycium barbarum* varieties [119], and convolutional neural network–long short-term memory (CNN-LSTM) analysis of petroleum derivatives in olive oil [120] show that variable selection and classifier choice can change a model’s sensitivity to batch variation. In fish and food-quality research [73], Raman, near-infrared, and hyperspectral technologies provide compositional, structural and spatial information, respectively. Models should therefore match task type with signal source. Reviews of Raman/SERS chemometrics [22] emphasize that preprocessing and validation cannot be separated from reference methods, and dairy-adulteration studies [46] further show that regression and classification answer different questions. For chip data, spectra, fluidic state, chip batch, detection position, and reference values should be recorded together to distinguish chemical changes from device changes.

### 7.2. Deep Learning and Multi-Source Data Fusion

Deep learning is suitable for high-dimensional images, time-series spectra, and multimodal data, but it requires more representative samples and independent testing [121]. A review of deep learning in near-infrared spectroscopy [76] discusses the application boundaries of regression models for food quality attributes. Source-classification tasks also indicate that origin classification must be tested on external regions or years. Japanese rice-variety studies combined fluorescence fingerprints, NIR, and machine learning [122], while edible-flower studies compared Vis-NIR and NIR for the prediction of active compounds [54]. For multimodal chips, feature-level fusion can combine the bulk compositional information of NIR with the molecular fingerprints of SERS, but repeated measurements from the same batch must not cause data leakage.

### 7.3. Model Generalization and Field Decision-Making

Model generalization depends on independent batches, independent devices, and independent matrices. Image-spectral fusion and attention networks for black-tea withering quality [69], multi-indicator attention models for freeze–thawed chicken breast [68], and multitask transformers for simultaneous evaluation of total volatile basic nitrogen (TVB-N) and total viable count (TVC) in chicken breast [86] show that one model can process several indicators, but also increases the problems of label synchronization and error propagation. Reviews of hyperspectral safety assessment for agricultural products and foods [123] remind us that a clear classification map does not mean that low-concentration targets have been quantified. Deep-learning classifiers for colony counting [124] likewise require explicit imaging conditions, counting standards, and real-sample boundaries. Field systems should output results, confidence, abnormality warnings, and applicable ranges, not only unexplained classes.

### 7.4. Algorithm Reporting Standards

Machine-learning reports should at least describe sample partitioning, preprocessing, feature selection, model hyperparameters, cross-validation, independent testing, evaluation metrics, and failed samples. Studies on oils, coffee, and dairy products have shown the utility of chemometrics in authenticity judgement, but sample size, label source, and validation design differ substantially across studies. More publications do not mean that models have achieved cross-laboratory capability. SERS drug classification models [125], automated textile sorting [114], and feature extraction with deep learning for COVID-19 screening [126] are non-food algorithmic references. They are useful for discussing feature selection, class imbalance, and handheld deployment, but cannot be used directly as food-application performance evidence. Quantitative SERS studies of single plasmonic nanogaps [127] and multivariate analysis of MnSOD fluctuation aptamer signals [128] are also non-food studies, but they provide methodological references for hotspot statistics, internal-standard design, and model stability. Figure 3 summarizes portable NIR, spatial/multispectral imaging, fluorescence-based artificial intelligence (AI) interpretation, and direct SERS readout routes, emphasizing that optical acquisition and downstream algorithmic interpretation should jointly serve field decision-making.

## 8. Validation in Real Foods

Real-food application is the decisive test of whether a microfluidic spectroscopic platform is valid. Compared with buffers or artificial standards, real foods introduce variation in target concentration, matrix composition, particle state, colour, viscosity, and processing history. This section primarily focuses on studies that combine microfluidics or paper-based microfluidics with spectroscopy or optical readout and that involve food or food-related samples. At the same time, a limited number of non-microfluidic food-matrix studies or non-food methodological studies are retained only as indirect supporting evidence, when they help explain matrix effects, spectral interference, target-substrate contact, enrichment principles, optical-interface design, or model-boundary problems. These studies are not treated as direct real-food validation of microfluidic-spectroscopic platforms. Instead, they are explicitly distinguished from direct application cases and are used only to complement the discussion of analytical mechanisms and translation constraints. The evaluation focuses on whether the sample is real, whether the chip performs a defined pretreatment or control function, whether the readout is connected to food judgement, and whether recovery, accuracy, repeatability, or external validation are reported. It should also be noted that genuinely validated on-chip food applications remain relatively limited compared with the much larger literature on food spectroscopy, SERS substrates, and general microfluidic sensing. This evidence gap is not only a limitation of the present review, but also an important indication that the field still needs more direct validation in real food matrices.

### 8.1. Pesticides, Heavy Metals and Small-Molecule Contaminants

Yan et al. developed a deep-learning-enhanced paper-based microfluidic fluorescence sensor for simultaneous detection of pesticides and metal ions in foods [82]. The abstract reported a reduction in image-processing time from minutes to milliseconds, a mean average precision at an intersection-over-union threshold of 0.50 (mAP50) of 0.995, an F1-score of 1, a classification accuracy of 91.3%, and detection limits of 5.84–11.70 nM for four targets. The value of this case lies in placing capillary transport, reaction-zone signals and model interpretation within one platform. The focus is not a single fluorescence peak but selectivity and readability under multi-target conditions. For nickel ions and ethylenediaminetetraacetic acid (EDTA) in foods, Yan et al. used a ratiometric fluorescence cellulose paper-based microfluidic chip together with an AI-assisted handheld sensor for direct reading [109]. Cai et al. constructed a molecularly imprinted, ratiometric fluorescence paper-based chip for sulfadiazine detection in real samples [84]. A multilayer paper-based microfluidic study used enzyme-inhibition impedance time-series spectroscopy to identify pesticide residues [83], demonstrating the compatibility of microfluidics with different signal routes. These results suggest that paper-based chips benefit from low-volume contact between reagent and sample, but quantitative credibility still depends on spiked recovery in real matrices and batch repeatability.

For lipid-oxidation-related small molecules, Lu et al. used phosphoric-acid-induced controllable nanoparticle aggregation to achieve ultrasensitive SERS detection of malondialdehyde in a microfluidic chip [85]. The chip did not simply carry a substrate; it controlled nanoparticle aggregation, analyte transport, and laser detection position. A NIR-SERS fusion model reported by Yan et al. further indicates that multispectral information can be used for quantitative detection of pesticide residues in foods, but model performance must be compared with single-spectrum models and independent prediction sets [34]. These studies together show that the logic of flowing sample, detection zone and spectral or colour output must be linked to real matrices and independent validation, and that detection limits for different targets should not be ranked directly across unrelated tasks.

### 8.2. Mycotoxins and Cereal Samples

Qi et al. designed a disposable microfluidic aptasensor for one-step real-time detection of aflatoxin B1 at sub-femtomolar levels in foods [129]. The study combines small volume, rapid reaction, and specific recognition but application to grains or nuts still requires evaluation of homogenization, extraction efficiency, and spatial contamination distribution. Zhu et al. combined solubility-difference-based microfluidic sorting with near-infrared detection in wheat, with the aim of first reducing matrix effects on mycotoxin prediction [80]. Veliz et al. reported a flow-through aptamer-SERS platform that places aflatoxin B1 (AFB1) enrichment, aptamer recognition, and Raman readout for food crops within a continuous-flow framework [97]. The two strategies represent different routes: the former emphasizes matrix separation and bulk spectra, whereas the latter emphasizes specific capture and enhanced fingerprints.

Guo et al. used short-wave infrared hyperspectral imaging to study the spatiotemporal distribution of aflatoxin B1 and total aflatoxins in peanut kernels [81]. Such results remind us that mycotoxins are not necessarily uniformly distributed, and that sampling and mixing before chip extraction directly affect final judgement. For grains and nuts, suitable microfluidic protocols should record sampling mass, grinding or homogenization, extraction volume, filtration loss, and representativeness of the detection region. Only when these steps are reproducible can spatial information from spectral models or quantitative results be meaningfully compared.

### 8.3. Foodborne Bacteria and Pathogens

Lin et al. combined SERS nanoprobes with a microfluidic dielectrophoresis device for online detection of single bacteria, including *Salmonella enterica* serotype Choleraesuis and Neisseria lactamica [101]. The work highlights the role of active enrichment and online positioning in single-cell signals. Zhuang et al. used a microfluidic paper-based analytical device that combined CRISPR/Cas recognition and SERS signals for the ultrasensitive detection of foodborne pathogens [99]. Wang et al. integrated NaYF4:Yb,Er@SiO2@Au structures inside a chip for near-infrared-excited SERS detection of *E. coli* [130]. These designs use nucleic acid specificity, nanostructure enhancement, and on-chip optical excitation to address low-abundance pathogen readout.

Jayan et al. reported a microfluidic-SERS platform with in situ nanoparticle synthesis for rapid *E. coli* detection in foods [107]. Huo et al. developed a flexible microfluidic co-recognition system coupled with magnetic enrichment, and silent SERS sensing for simultaneous analysis of multiple bacteria in foods [108]. Dogan et al. used a capillary-driven microfluidic chip and SERS for *E. coli* enumeration [98]. These examples show that magnetic enrichment, capillary transport and in situ nanoparticle generation can reduce external equipment, but they also introduce new variables, including substrate batch variation, cell-capture efficiency and hotspot distribution. Application studies should report negative foods, related bacterial species, real samples, and reference culture or PCR results.

Xie et al. proposed a channel–digital hybrid microfluidic platform for on-site multiplex detection of foodborne pathogens [100]. Li et al. constructed a portable multispectral diffraction microfluidic system that purified pathogenic fungi on chip and collected diffraction fingerprints, achieving 98.7% recognition accuracy across five fungal classes and similar particles [110]. These two studies represent multiplex reaction and multispectral morphological-recognition routes, respectively. They show that the application value of microfluidic spectroscopic systems is not only stronger single peaks, but also the organization of sample splitting, target purification, and portable judgement into a complete workflow [131].

### 8.4. Veterinary Drugs, Dyes and Food-Quality Indicators

Fan et al. used a chewing-gum-based viscoelastic SERS sensor to screen malachite green contamination on live fish skin [132], aiming to reduce complex sampling and improve surface-contact convenience. Salman et al. used nitrogen-doped carbon quantum dots as a fluorescence sensor for quantifying Allura Red in beverages [133], illustrating selective fluorescence analysis in strongly coloured matrices. These two studies are retained here as indirect food-matrix-supporting evidence rather than direct microfluidic-spectroscopic validation, because they do not represent fully integrated microfluidic platforms. Nevertheless, they help show that food surfaces and beverage matrices can alter signal formation. Real applications therefore cannot rely only on aqueous standard curves.

**Figure 4:**
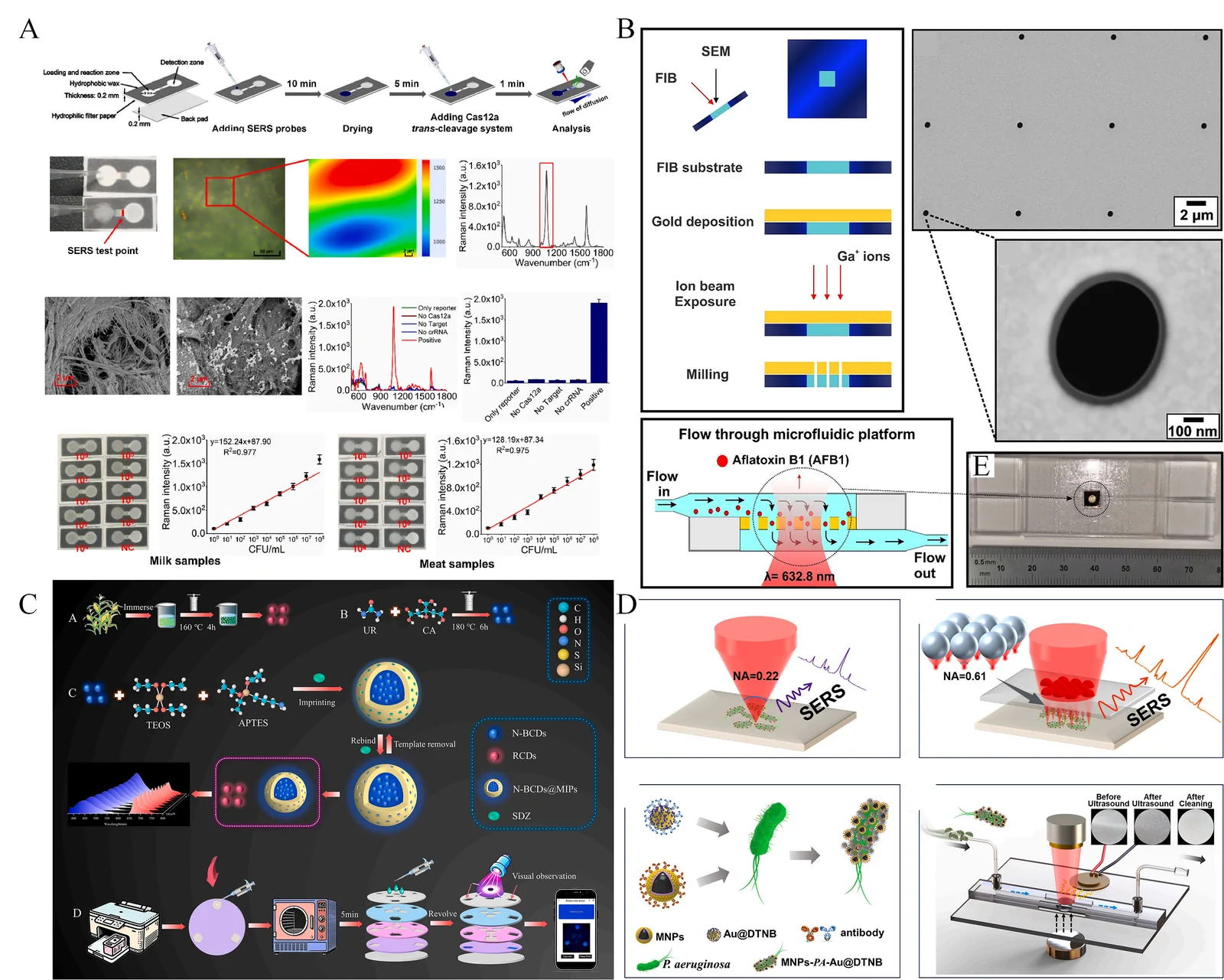
Representative real-food applications of microfluidic spectroscopic analysis. (**A**) Integrated SERS and nucleic acid-assisted workflow for bacterial detection in food matrices. (**B**) Flow-through microfluidic SERS platform based on nanostructured substrates for mycotoxin analysis. (**C**) Fluorescent/molecularly imprinted sensing strategy for contaminant recognition and portable readout. (**D**) Representative optical and SERS configurations for pathogen, contaminant, and quality-related food analysis.

Hydrogen peroxide, veterinary drugs, and small-molecule contaminants in foods also require attention to reaction selectivity and sample pretreatment. Magnetic nanozyme-based SERS-colorimetric dual-mode detection [32] is retained as indirect supporting evidence for cross-checking spectral and colour outputs. Aptamer-SERS detection of lysozyme on food-contact surfaces [41] indicates that the boundary between surface sampling and contamination-residue judgement must be defined. Early SERS detection of acetamiprid in foods [134] provides a basic case for target-substrate contact. These studies are used to supplement the discussion of matrix effects, surface sampling, and target-substrate contact, but they are not counted as direct microfluidic–spectroscopic food validation unless a microfluidic component is explicitly involved. When such strategies are coupled with microfluidic platforms, the workflow from sample entry to target transfer, reaction/enrichment, spectral readout, and judgement should be recorded step by step, with explicit notation of which steps remain off chip. Figure 4 summarizes representative real-food application cases, covering foodborne pathogens, mycotoxins, food contaminants, and quality/oxidation indicators. It illustrates how different tasks organize sample entry, target transfer, reaction/enrichment, spectral readout, and result judgement into a complete workflow. Table 4 complements the case discussion by listing target analytes, sample matrices, microfluidic functions, spectroscopic routes, performance metrics, and evidence levels in representative real-food applications.

## 9. Standardization, Translation and Future Research Directions

For microfluidic spectroscopic food detection to move from research devices to comparable methods, descriptions of samples and chips must first be standardized. Research progress on the vibrational spectroscopic detection of mycotoxins in cereals and cereal products indicates that sample source, contamination level, spectral range, preprocessing, reference method and model validation must be reported together; otherwise, R, RMSE and detection limits cannot be compared across studies [135]. Food-detection reviews and spectroscopic overviews also indicate that laboratory models and field methods differ in equipment, sample state, and operator dependence [136]. Research articles should therefore record at least channel material, width and height, flow rate or capillary time, sample volume, enrichment steps, light-source and detector parameters, spectral preprocessing, and independent testing protocols.

Second, an external validation system for real foods should be established. Studies of paper-based and polymeric lab-on-a-chip devices show that low cost does not mean low variation [137], and nucleic acid analysis chips show that lysis, purification, amplification and interpretation can each become limiting steps [138]. For NIR, Raman, SERS, fluorescence and terahertz platforms, the effects of sample thickness, water content, temperature, colour, particles, and native fluorescence should be assessed separately. Cross-food-matrix generalization cannot be demonstrated only by random sample splitting, but should reserve external samples by origin, batch, variety, processing condition or device. For SERS, substrate-batch hotspots, surface contamination, nanomaterial aggregation, and competitive adsorption should be quality-control items. For hyperspectral imaging and machine learning, spatially adjacent pixels or repeated measurements from the same batch must not be placed simultaneously into training and test sets. The review by Wu et al. on nanostructured optical and electrochemical biosensing further emphasizes that interface design among materials, devices and food-safety scenarios is central to platform translation [139].

Third, future systems should gradually establish traceable data structures. Each spectrum or image should be linked to sample identifier, food matrix, processing steps, chip batch, channel state, temperature, flow rate, optical position, and reference value. Abnormal data, failed experiments, and boundary samples should not simply be removed. Cross-device model transfer can draw on spectral calibration transfer and multi-source fusion, but must demonstrate that post-transfer errors still satisfy practical decision requirements. Portable systems should report concentration or class, confidence, applicable range and abnormality cause. For regulatory or industrial use, raw spectra and calibration versions should be retained so that historical results remain traceable after model updates [140].

Finally, research on microfluidics and spectroscopy should shift from integrating more functions to completing verifiable tasks with fewer functions. If a sample only needs filtration and a fixed optical path, complex multivalve structures are unnecessary. If the target requires specific capture, capture efficiency and nonspecific adsorption should be solved first. If a model depends on multimodal data, calibration and missing-data rules for each channel must be established synchronously. Chips do not need to be fully universal for all foods. A bibliometric survey of olive-oil spectroscopic authentication by El Morabit et al. shows that research hotspots are shifting toward artificial intelligence and multi-source data fusion [141], but a larger literature does not mean that models have achieved cross-laboratory capability. A more realistic direction is to build replaceable modules around typical matrices, such as high-fat, high-protein, highly pigmented or highly particulate foods, and connect them to portable spectrometers and algorithms through standardized interfaces. Only then can time, cost, accuracy, portability, automation, and cross-matrix capability be converted into comparable engineering metrics.

Overall, the research value of microfluidics combined with spectroscopy lies in incorporating the complexity of food samples into the detection system itself. The chip controls sample state and target position, spectroscopy provides compositional, structural or reaction information, and the model performs quantification, classification, and abnormality judgement. Translationally promising systems should demonstrate controllable sample-processing loss, stable spectral readout, external model validity, chip-to-chip consistency and completion of the whole sample-to-result workflow at reasonable cost in real foods.

## 10. Conclusions

The main value of microfluidics integrated with spectroscopy is that it incorporates state control of complex food samples into the detection process, allowing filtration, homogenization, separation, enrichment, reaction and optical readout to operate around a single analytical task. Near-infrared spectroscopy and hyperspectral imaging are suitable for characterizing bulk composition, spatial distribution and quality changes in foods. Raman/SERS is suited to molecular fingerprints and specifically enhanced signals. Fluorescence, colorimetric, terahertz and impedance time-series spectroscopies can provide different field-readout routes depending on reaction system, sample state and equipment conditions. Microfluidics does not automatically remove spectral backgrounds, substrate batch effects, or model bias. Whether it improves detection performance depends on whether the chip truly reduces matrix interference, stabilizes optical path length, improves target-transfer efficiency, and is supported by recovery, detection limits, precision, analysis time, and external validation. Existing studies also show that food-safety detection and food-quality evaluation should not use the same chip logic. Low-concentration pesticides, mycotoxins, heavy metals, and pathogens usually require capture, enrichment, or selective reactions, whereas authenticity, freshness, maturity, and oxidation depend more on representative sampling, continuous-state calibration, and cross-batch modelling. Future research should move from adding chip functions toward establishing verifiable sample-to-result workflows, clearly specifying which steps are completed on chip and which still rely on external pretreatment, while systematically reporting chip dimensions, flow rate, sample volume, optical parameters, batch variation, failed samples, and independent testing. Only when microfluidic structures, spectral signals, and decision models form a reproducible, traceable, and cost-acceptable loop in real foods will these methods have a foundation for transition from laboratory prototypes to field detection and scaled application.

## Figures and Tables

**Table 1:** Food targets, matrices, analytical bottlenecks, and microfluidic intervention points.

Problem Axis	Representative Targets	Typical Food Matrices	Analytical Bottleneck	Microfluidic Intervention	Literature Anchors
Biological hazards	Foodborne pathogens, bacterial toxins, fungal contamination	Milk, meat, produce, grains and complex food extracts	Low abundance, complex background, multiplex detection	Membrane concentration, magnetic enrichment, capillary transport, on-chip nucleic acid processing	[42,79,80,81,82,83,84,85] as methodological examples
Mycotoxins	AFB1 and wheat/grain mycotoxins	Wheat, grains, nuts and food crops	Trace targets, particle heterogeneity, solubility differences	Solubility-based sorting, flow-through recognition, aptamer capture	[80,81]
Chemical hazards	Pesticides, heavy metals, antibiotics, illegal additives	Produce, beverages, grains, and food extracts	Multiple targets, matrix interference, field quantification	Multiplex reaction zones, compartmental enrichment, image-based readout	[34,35,82,83]
Veterinary drugs and antibiotics	Sulfonamides, antibiotics and related residues	Dairy, meat, and real-food samples	Selectivity, low sample volume, rapid quantification	Paper microfluidics, molecular imprinting, ratiometric correction	[29,33,84]
Authenticity and adulteration	Dilution, substitution, origin/species differences and fraud markers	Edible oils, honey, dairy, tea, spices and liquid foods	Weak multicomponent differences; cross-batch and cross-origin generalization	Compartmental sampling, continuous-flow mixing, automated spectral acquisition	Spectroscopic model evidence: [46,49,50,51,52,53,55]; chip role as translation direction
Quality and freshness	MDA, lipid oxidation, spoilage and processing changes	Edible oils, meat, dairy, beverages, and produce	Dynamic changes, quantitative reproducibility, process monitoring	Micromixing, controlled aggregation, online sampling and, continuous monitoring	[62,63,67,73,85,86] microfluidic SERS

**Table 4:** Quantitative performance and evidence levels of representative microfluidic–spectroscopic applications in food safety and quality detection.

Application Category	Target/Matrix	Microfluidic or Supporting Role	Readout	Quantitative Analytical Performance	Validation/Reporting Note	Evidence Level	Representative References
Foodborne pathogens	Bacteria and pathogenic fungi; milk, meat, food samples, or food-related particles	Paper/continuous-flow chips, CRISPR reaction, magnetic enrichment, capillary transport, or on-chip purification	SERS, colorimetry, or multispectral diffraction	Pathogenic fungi recognition accuracy: 98.7% [110]; other LOD/time values: NR	Report negative matrices, related species, real samples, and culture/PCR comparison where available	Direct or food-related microfluidic-spectroscopic evidence	[98,99,100,107,108,110,130]
Mycotoxins	AFB1 and wheat/grain mycotoxins; wheat, grains, nuts, and food crops	Disposable microfluidic aptasensing, flow-through aptamer recognition, or solubility-difference-based sorting	SERS or NIR	AFB1 LOD: 10 ppb; analysis time: ca. 10 min [97]; sub-femtomolar AFB1 detection reported [129]; other range/recovery values: NR	Screening interpretation requires matrix-specific recovery, calibration and confirmatory validation	Direct food or food-related microfluidic-spectroscopic evidence	[80,97,129]
Pesticides/heavy metals	Pesticides, Ni ions, and EDTA; food samples	Paper-based reaction zones, ratiometric correction, low-volume capillary transport, or multilayer paper channels	Fluorescence imaging, AI-assisted reading, or impedance time-series	LOD: 5.84–11.70 nM for four targets; mAP50: 0.995; F1-score: 1; classification accuracy: 91.3% [82]; other values: NR	Quantitative credibility depends on recovery in real matrices and batch repeatability	Direct paper-based microfluidic-optical food evidence	[82,83,109]
Veterinary-drug residues	Sulfadiazine; actual food samples	Molecularly imprinted paper-based chip with confined reagent contact and visual reaction zone	Ratiometric fluorescence	LOD, linear range, recovery, and precision: NR	Actual food-sample use is retained; full performance reporting should be checked from the cited article	Direct paper-based microfluidic-optical food evidence	[84]
Quality/lipid oxidation	Malondialdehyde; edible oils, and fat-rich food systems	Microfluidic mixing and controllable nanoparticle aggregation in a defined detection zone	SERS	LOD, calibration range, recovery, repeatability, and time: NR	Performance values should be interpreted within lipid-oxidation matrices rather than across unrelated targets	Direct microfluidic-SERS food-quality evidence	[85]
Indirect food-matrix support	Minimally processed foods, H2O2 in foods, lysozyme on food-contact surfaces, malachite green on fish skin, Allura Red in beverages, and acetamiprid in foods	No clear integrated microfluidic component; used to explain matrix effects, surface sampling, target-substrate contact and field-readable signals	SERS, fluorescence or SERS-colorimetry	Matrix response or screening performance reported in the cited studies; not used as microfluidic validation metrics	Retained only as background support and not counted as direct real-food microfluidic validation	Indirect supporting evidence	[30,32,41,132,133,134]
Indirect methodological support	Non-food model systems or optical-interface studies	Used to explain enrichment, optical focusing, signal stability, or detection-zone design	Microfluidic SERS or optical readout	Methodological performance only; not compared directly with food-matrix applications	Discussed in architecture/interface sections rather than as direct food validation	Non-food methodological evidence	[91,94]
Authenticity/adulteration background	Oils, honey, dairy, tea, coffee, spices, and other food matrices	Supports spectral modelling and cross-batch validation needs; microfluidics is discussed as a translation direction	NIR, FTIR, Raman or hyperspectral imaging with chemometrics	Classification accuracy, RMSE, prediction error or external validation metrics: study-dependent	Used to define modelling requirements, not as direct microfluidic validation	Indirect spectroscopic-model evidence	[46,49,50,51,52,53,55]

Note: NR, not reported or not applicable. Direct and indirect evidence are separated to avoid treating supporting food-matrix or non-food methodological studies as direct microfluidic validation. Quantitative metrics should be compared only within similar target classes and matrices.

## Data Availability

No new data were created or analysed in this study. Data sharing is not applicable to this article. The literature information used for discussion is available in the cited publications, and the comparison with previous reviews is provided as Supplementary Table S1.

## Supplementary Materials

The following supporting information can be downloaded at: https://www.mdpi.com/article/10.3390/foods15183171/s1, Table S1. Comparison between this review and representative previous reviews.
