# Supplementary material for: Microfluidics-Integrated Spectroscopic Technologies for Food Safety and Quality Assessment: From Complex-Matrix Processing to On-Site Decision-Making
Source: Foods. 2026 Sep 8;15(18):3171. doi: 10.3390/foods15183171 (PMC13605163; doi:10.3390/foods15183171)
Supplement: Supplementary file 1 [file foods-15-03171-s001.zip › foods-4500452-supplementary.pdf]

Table S1 Comparison between this review and representative previous reviews.

Previous review(s) in this manuscript	Main focus	Aspect less emphasized relative to this review	How the present review extends the discussion
Food spectroscopy reviews [71], [73], [74], [75]	Spectroscopic evaluation of edible oils, milk/dairy hazards, agri-food quality and safety, and NIR/hyperspectral modelling.	These reviews mainly discuss spectroscopy as the analytical route, whereas chip-level sample handling and detection-zone control are not their central focus.	Links food spectroscopy with microfluidic sample entry, pretreatment, enrichment, optical-interface design and field-oriented judgement.
Food Raman/SERS reviews [2], [4], [6], [22]	SERS sensing, Raman/SERS fingerprints, quantitative food-safety detection and chemometric interpretation.	The emphasis is mainly on enhancement materials, spectral signals or data modelling, with less systematic comparison of microfluidic workflow functions.	Connects SERS performance with target transfer, hotspot formation, matrix clean-up, chip architecture and real-food validation.
Target- or substrate-focused SERS reviews [5], [76], [115]	Mycotoxin SERS detection and flexible or hydrogel-based SERS substrates for food analysis.	These reviews focus on specific target classes or substrate materials rather than comparing multiple spectroscopic routes under a shared microfluidic framework.	Places substrate stability, diffusion, anti-matrix capability and target recognition within a broader sample-to-decision workflow.
Portable SERS and lateral-flow sources [9], [107]	Portable Raman/SERS instruments and SERS-enabled lateral-flow strategies for food-contaminant screening.	These studies are highly relevant to point-of-need detection, but they do not fully address how different microfluidic formats should be classified by evidence level.	Separates portability from verifiable sample-state control and distinguishes direct food validation from methodological references.
Raman/SERS microfluidic pathogen review [39]	Raman spectroscopy-based microfluidic platforms for foodborne pathogen detection.	The scope is centered on foodborne pathogens rather than broader food-safety and quality tasks.	Extends the chip-spectroscopy logic from pathogen detection to multiple food-safety and quality tasks.

Lab-on-a-chip food-safety reviews [137], [138]	Polymeric/paper-based lab-on-a-chip devices and nucleic-acid analysis chips for food safety.	These reviews emphasize lab-on-a-chip systems, but comparison among different spectroscopic and optical readout routes is not their main objective.	Couples lab-on-a-chip sample processing with NIR, Raman/SERS, fluorescence, colorimetry, terahertz, and impedance time-series readouts.
Terahertz food-analysis review [7]	Terahertz solution-phase sensing, metamaterial-enhanced microfluidics and functional-material integration for food analysis.	The discussion is centered on terahertz sensing rather than cross-modality comparison with established optical and vibrational techniques.	Positions terahertz sensing within a broader modality comparison and clarifies its current maturity relative to other food-analysis routes.
Machine-learning food spectroscopy reviews [10], [75]	Deep-learning-enhanced spectroscopy, NIR/hyperspectral regression, model interpretation and food-quality assessment.	These reviews mainly emphasize modelling strategies, while sample processing, chip-to-model traceability and real-food validation boundaries are less central.	Links preprocessing and model validation to chip function, matrix variation, sample batch and on-site decision-making.
